# Supplementary figures and images for: Metabolic and signalling network maps integration: application to cross-talk studies and omics data analysis in cancer
Source: BMC Bioinformatics. 2019 Apr 18;20(Suppl 4):140. doi: 10.1186/s12859-019-2682-z (PMC6471697; doi:10.1186/s12859-019-2682-z)

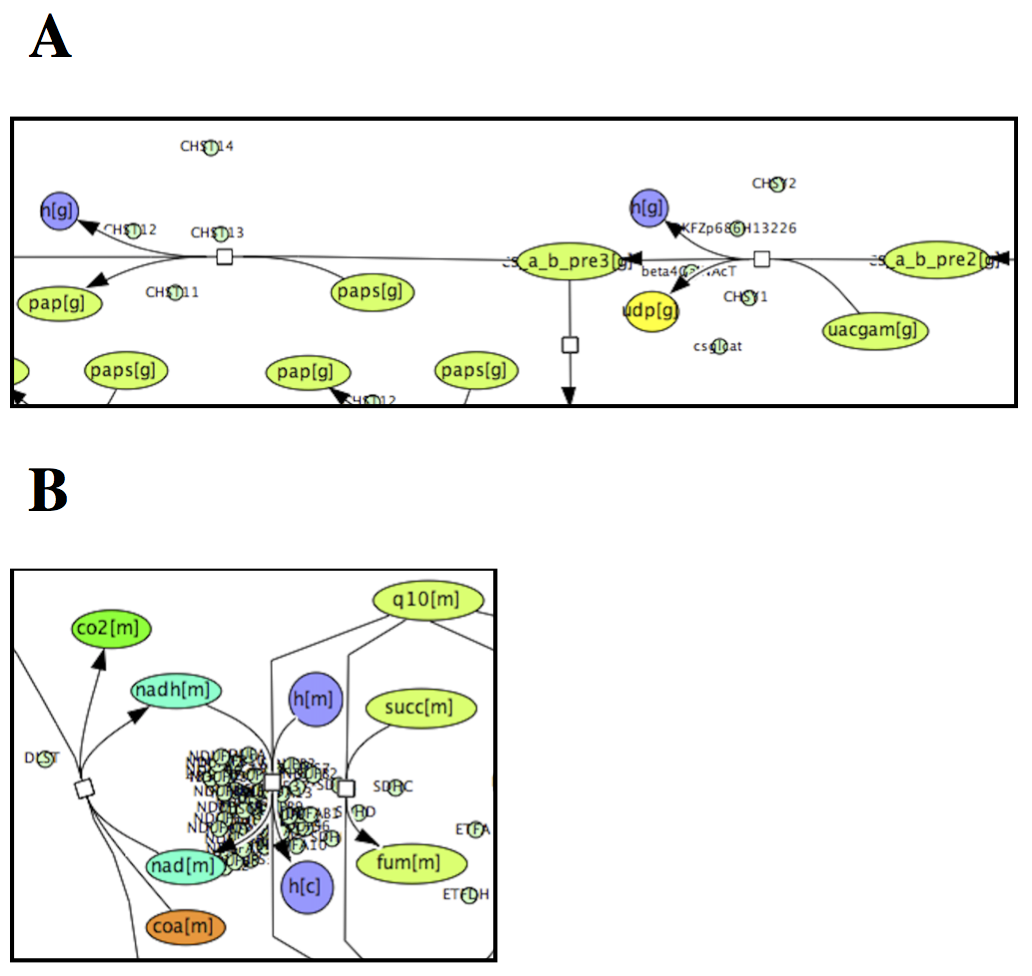

Supplement: Supplementary file 2 — Protein nodes display on the ReconMap 2.0 canvas. (A) Dispersed distribution of protein nodes around reactions, (B) Case of dense protein nodes distribution around a reaction, implicating 46 proteins. Protein nodes are represented by pale green circles. (TIFF 357 kb) [file 12859_2019_2682_MOESM2_ESM.tiff]

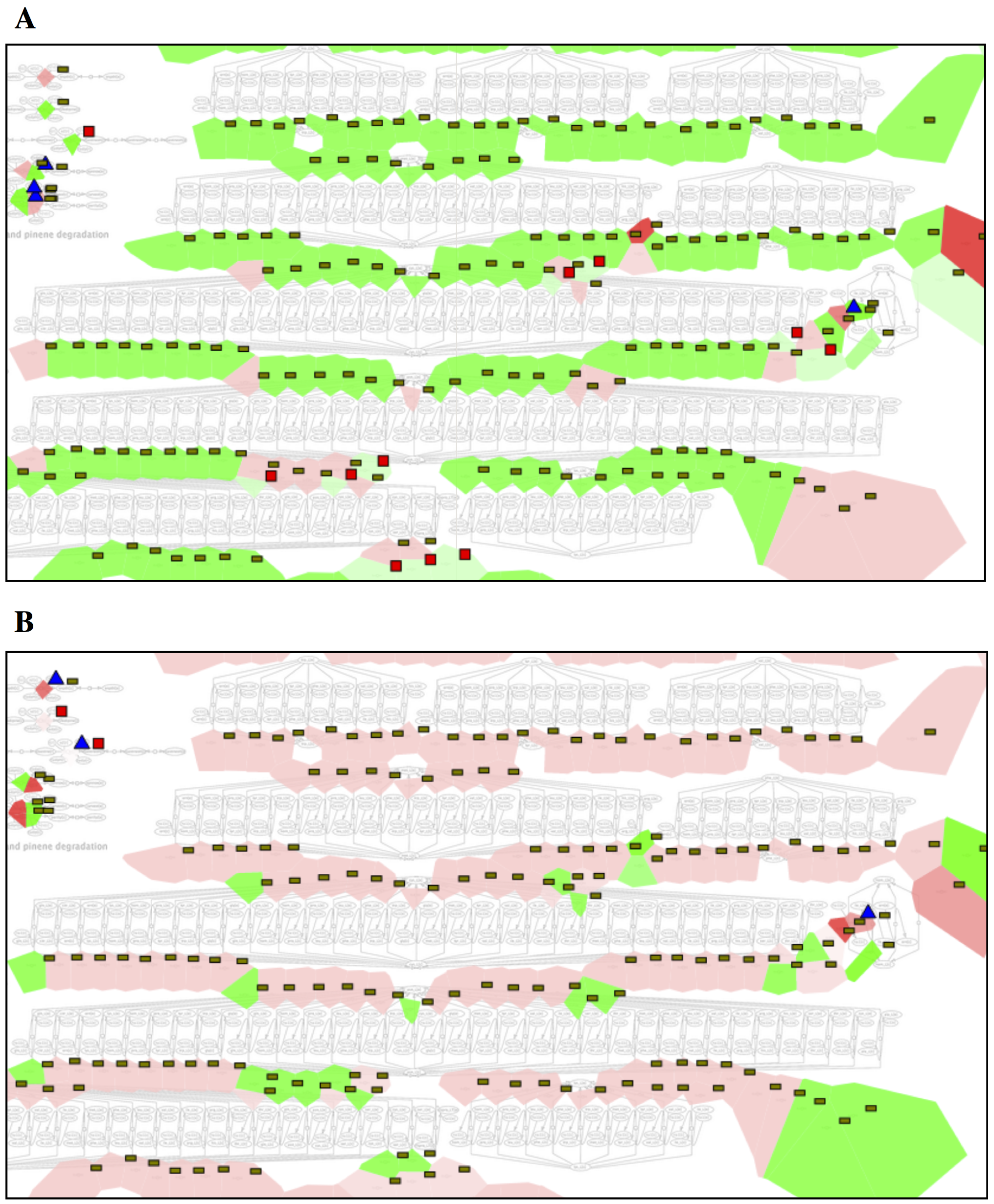

Supplement: Supplementary file 9 — Ovarian cancer multi-omics data visualisation on ReconMap 2.0: zoomed on nucleotide transport metabolic pathway. Two ovarian cancer subtypes are compared: Immunoreactive (top), Proliferative (bottom) Patches using the map staining function represent the average expression level of the corresponding genes (under-expressed in green and over-expressed in red). Barplots indicate the copy number state (red means at least 2 copy number). Glyphs shown as blue triangles are viewed near genes possessing mutations. (TIFF 3250 kb) [file 12859_2019_2682_MOESM9_ESM.tiff]

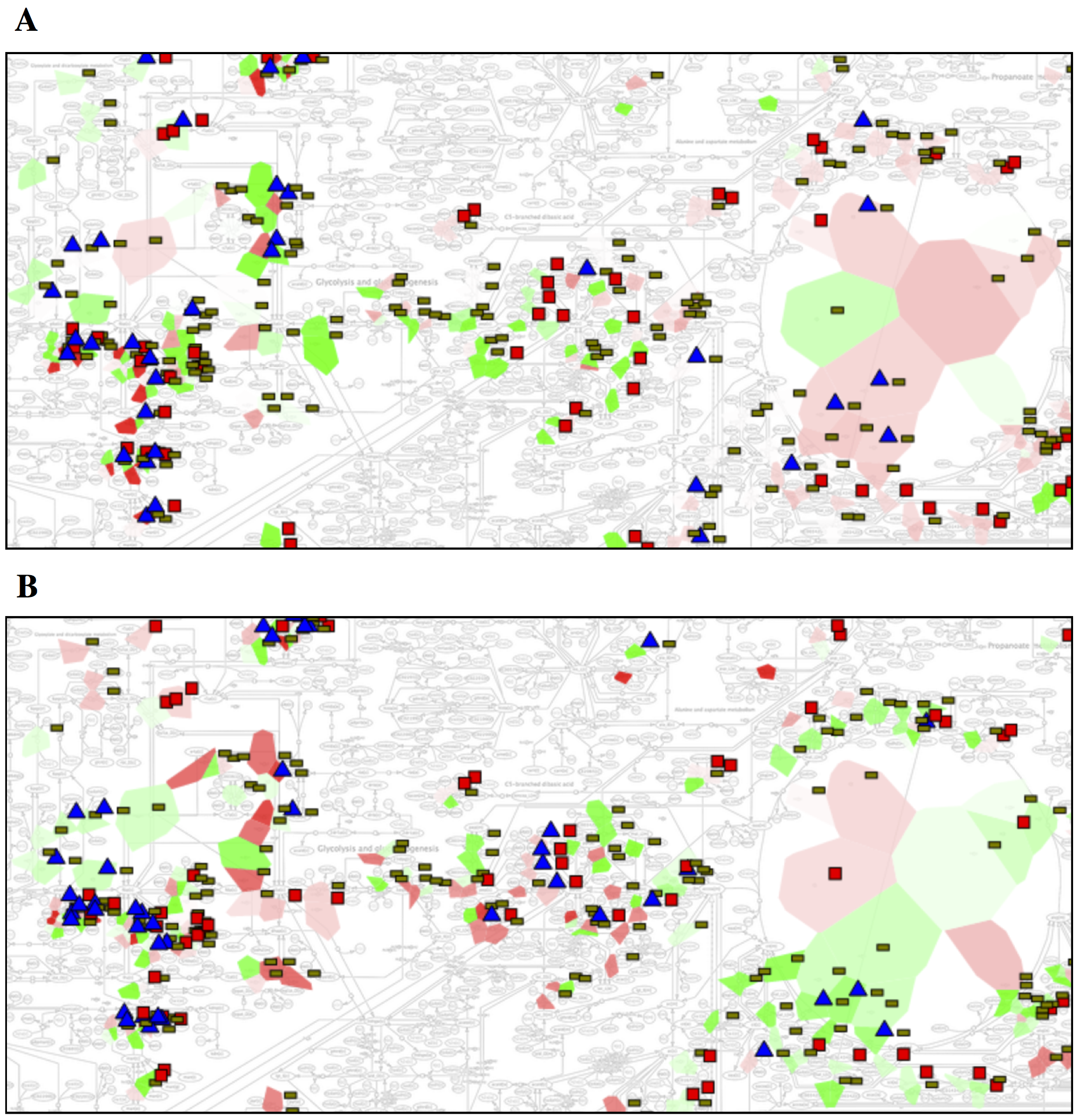

Supplement: Supplementary file 10 — Ovarian cancer multi-omics data visualisation on ReconMap 2.0: zoomed on energy metabolism pathways. Two subtypes are present: Immunoreactive (top), Proliferative (bottom). Patches using the map staining function represent the average expression level of the corresponding genes (under-expressed in green and over-expressed in red). Barplots indicate the copy number state (red means at least 2 copy number). Glyphs shown as blue triangles are viewed near genes possessing mutations. (TIFF 3412 kb) [file 12859_2019_2682_MOESM10_ESM.tiff]

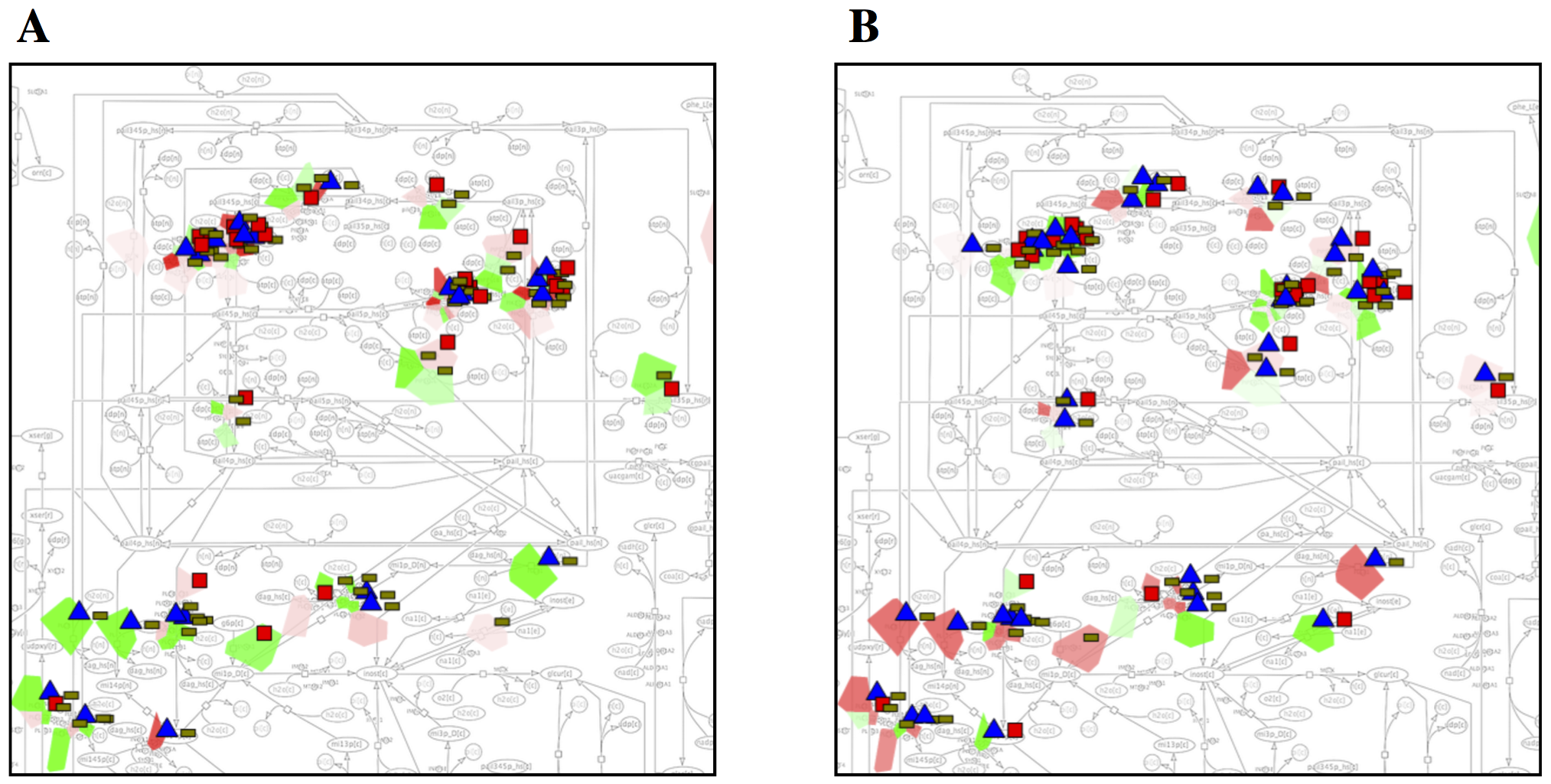

Supplement: Supplementary file 11 — Ovarian cancer multi-omics data visualisation on ReconMap 2.0: zoomed on inositol phosphate metabolic pathway. Immunoreactive (A) and Proliferative (B) subtypes are compared. Patches using the map staining function represent the average expression level of the corresponding genes (under-expressed in green and over-expressed in red). Barplots indicate the copy number state (red means at least 2 copy number). Glyphs shown as blue triangles are viewed near genes possessing mutations. (TIFF 1338 kb) [file 12859_2019_2682_MOESM11_ESM.tiff]

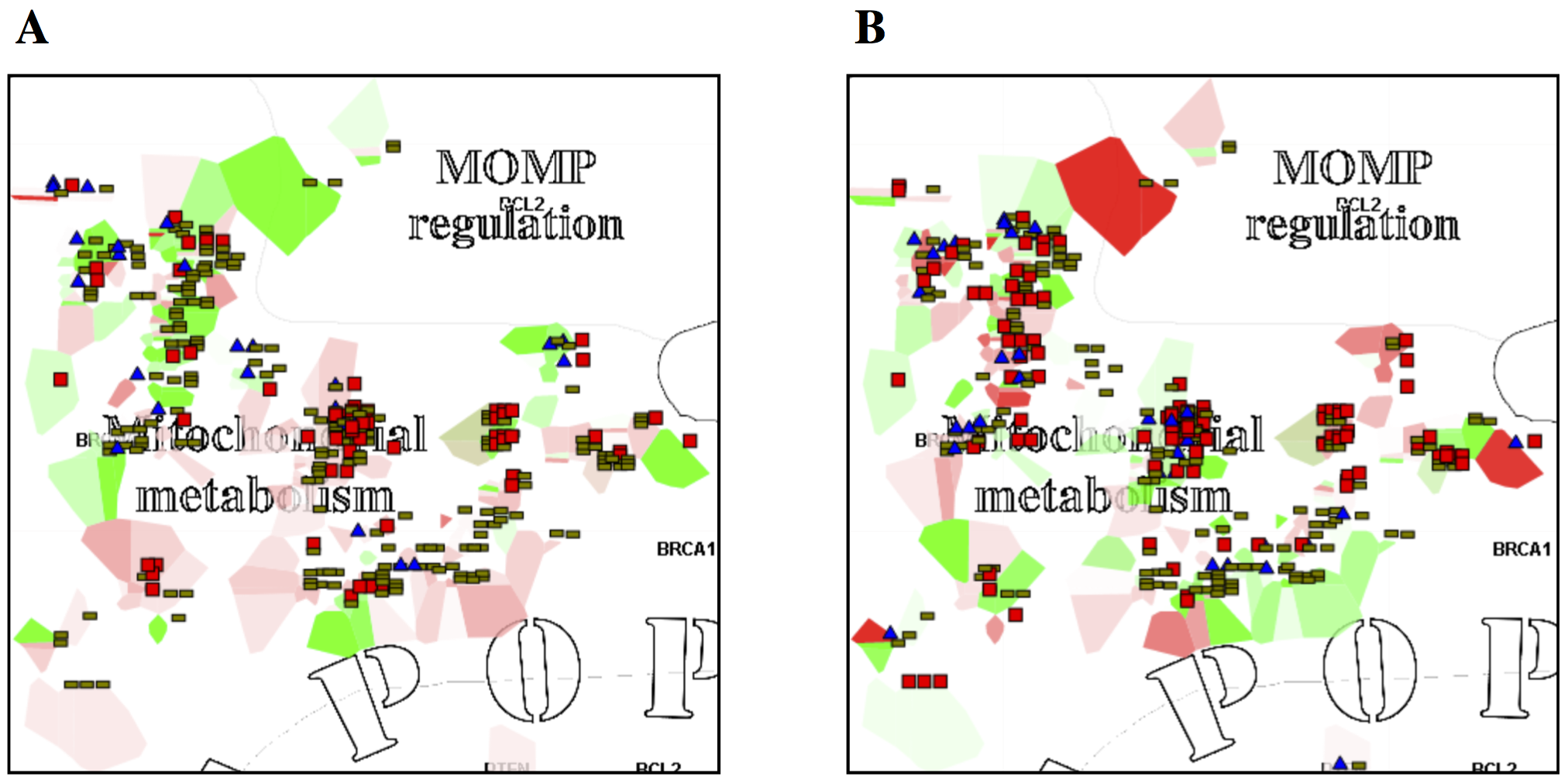

Supplement: Supplementary file 12 — Ovarian cancer multi-omics data visualisation on ACSN: zoomed on mitochondrial metabolism signalling pathway. Immunoreactive (A) and Proliferative (B) subtypes are compared. Patches using the map staining function represent the average expression level of the corresponding genes (under-expressed in green and over-expressed in red). Barplots indicate the copy number state (red means at least 2 copy number). Glyphs shown as blue triangles are viewed near genes possessing mutations. (TIFF 935 kb) [file 12859_2019_2682_MOESM12_ESM.tiff]

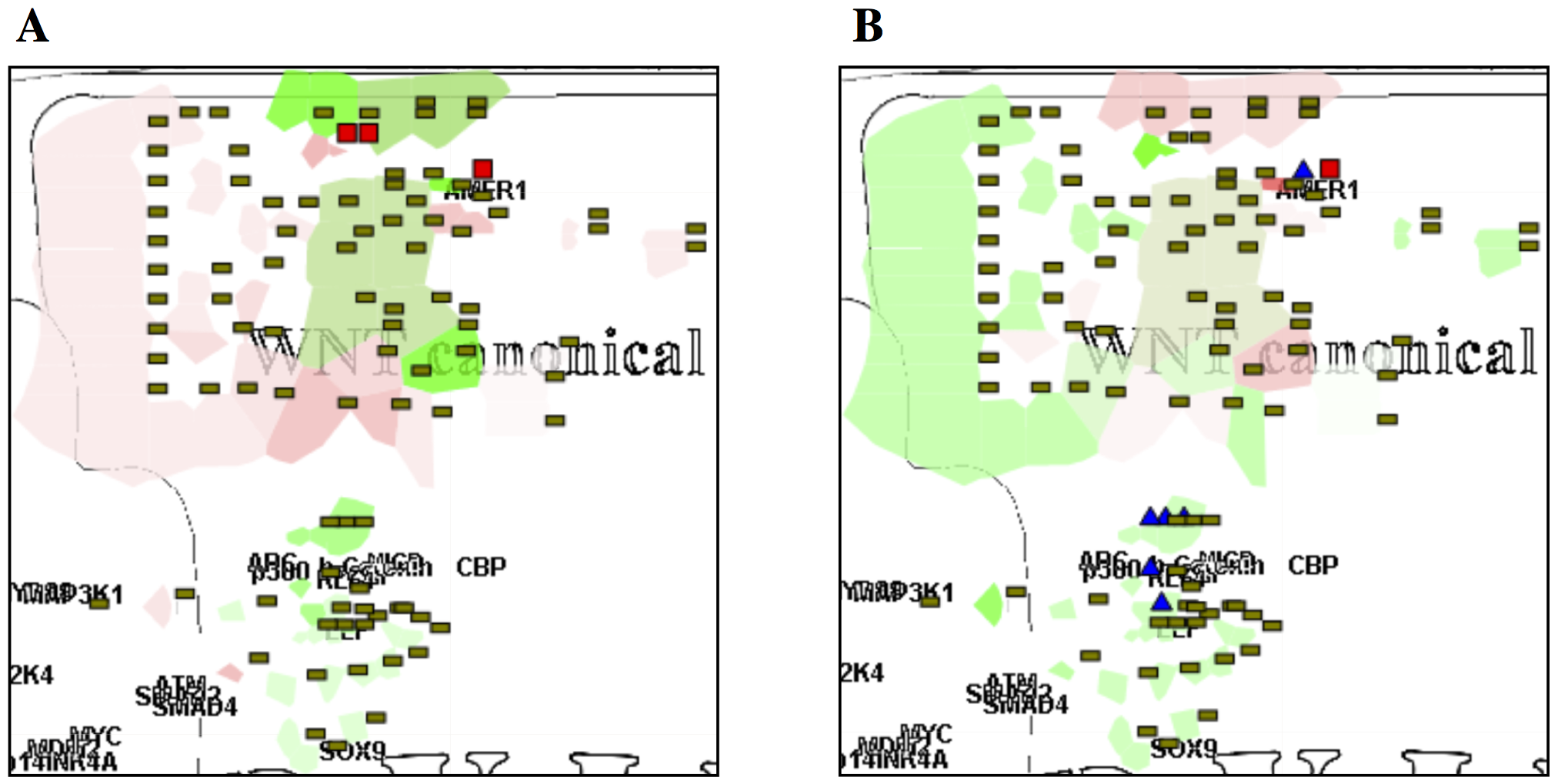

Supplement: Supplementary file 13 — Ovarian cancer multi-omics data visualisation on ACSN: zoomed on WNT canonical signalling pathways. Immunoreactive (A) and Proliferative (B) subtypes are compared. Patches using the map staining function represent average the expression level of the corresponding genes (under-expressed in green and over-expressed in red). Barplots indicate the copy number state (red means at least 2 copy number). Glyphs shown as blue triangles are viewed near genes possessing mutations. (TIFF 763 kb) [file 12859_2019_2682_MOESM13_ESM.tiff]

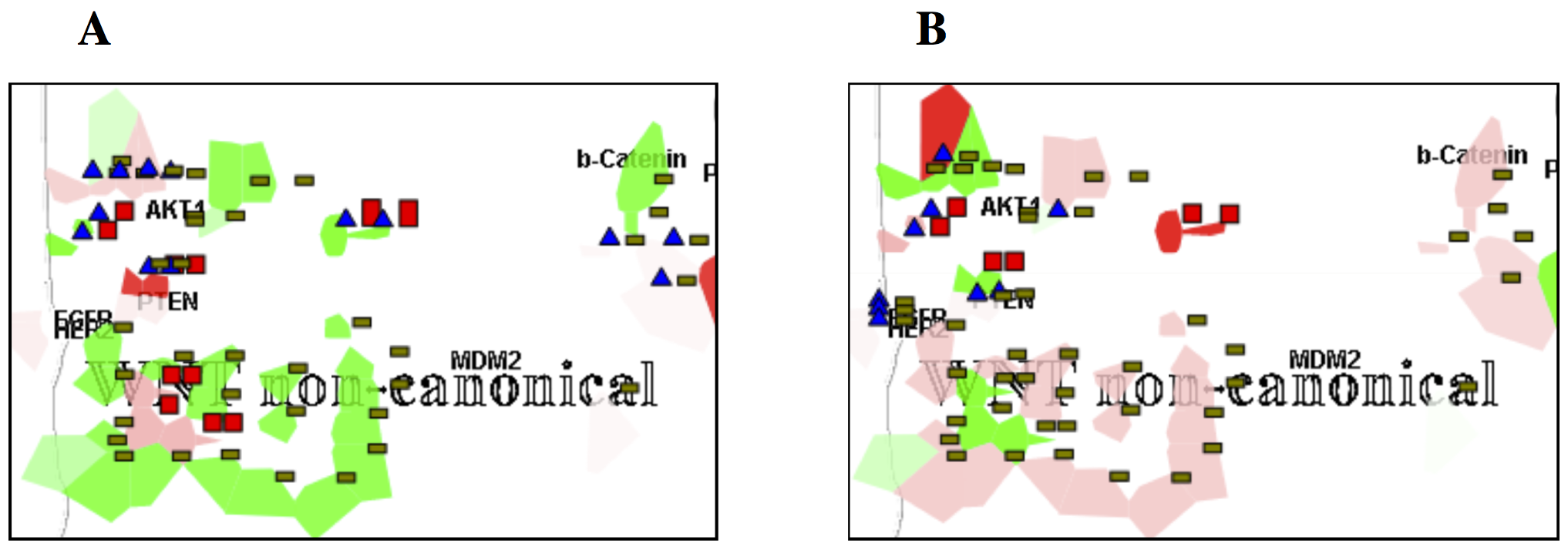

Supplement: Supplementary file 14 — Ovarian cancer multi-omics data visualisation on ACSN: zoomed on WNT non-canonical signalling pathway. Immunoreactive (A) and Proliferative (B) subtypes are compared. Patches using the map staining function represent the average expression level of the corresponding genes (under-expressed in green and over-expressed in red). Barplots indicate the copy number state (red means at least 2 copy number). Glyphs shown as blue triangles are viewed near genes possessing mutations. (TIFF 534 kb) [file 12859_2019_2682_MOESM14_ESM.tiff]
