# Supplementary material for: Metabolic and signalling network maps integration: application to cross-talk studies and omics data analysis in cancer
Source: BMC Bioinformatics. 2019 Apr 18;20(Suppl 4):140. doi: 10.1186/s12859-019-2682-z (PMC6471697; doi:10.1186/s12859-019-2682-z)
Supplement: Supplementary file 5 — Crosstalk between ACSN modules and ReconMap 2.0 metabolic pathways. Using the composition of each module and subsystem (Additional files 3 and 4) and the list of the 252 shared proteins between both maps, modules and subsystems having common proteins were linked together. Thus, a crosstalk between both maps has been uncovered with the number of shared proteins showed here. *Modules and subsystems in bold are the result of an Enrichment Analysis of the 252 common proteins between ACSN and ReconMap 2. (DOCX 16 kb) [file 12859_2019_2682_MOESM5_ESM.docx]

| **ACSN Modules** | **Number of shared proteins in ACSN modules** | **ReconMap 2.0 subsystem** | **Number of shared proteins in ReconMap 2.0 subsystems** |
| --- | --- | --- | --- |
| **MITOCH_METABOLISM*** | 186 | Propanoate_metabolism;Transport_golgi_apparatus;Valine_leucine_and_isoleucine_metabolism;Glutamate_metabolism;C5-branched_dibasic_acid_metabolism,Fatty_acid_synthesis;**Citric_acid_cycle***;Vitamin_C_metabolism;Nucleotide_interconversion;Glycine_serine_alanine_and_threonine_metabolism;Galactose_metabolism;**Pentose_phosphate_pathway***;Miscellaneous,Exchange/demand_reaction;Aminosugar_metabolism;Glyoxylate_and_dicarboxylate_metabolism;Lysine_metabolism;Transport_extracellular;Fatty_acid_oxidation;Glutathione_metabolism;**Fructose_and_mannose_metabolism***;Transport_mitochondrial;Pyrimidine_synthesis;Heme_synthesis;Butanoate_metabolism;Pyruvate_metabolism;D;alanine_metabolism;**Glycolysis/gluconeogenesis***;Transport_peroxisomal;**Oxidative_phosphorylation***;ROS_detoxification;Bile_acid_synthesis;Methionine_and_cysteine_metabolism;Purine_synthesis;Arginine_and_Proline_Metabolism;Heme_degradation | 245 |
| **APOPTOSIS_GENES*** | 46 | Transport_golgi_apparatus;Glutamate_metabolism;CoA_catabolism;**Citric_acid_cycle***;Glyoxylate_and_dicarboxylate_metabolism;Glycine_serine_alanine_and_threonine_metabolism;**Inositol_phosphate_metabolism***;**Pentose_phosphate_pathway***;Aminosugar_metabolism;Miscellaneous;Transport_extracellular;Propanoate_metabolism;**Fructose_and_mannose_metabolism***;Heme_synthesis;Exchange/demand_reaction;Pyruvate_metabolism;**Glycolysis/gluconeogenesis***;ROS_detoxification;Glutathione_metabolism;Methionine_and_cysteine_metabolism;**Oxidative_phosphorylation***;CoA_synthesis;Heme_degradation | 56 |
| WNT_NON_CANONICAL | 23 | Transport_lysosomal;Exchange/demand_reaction;Phosphatidylinositol_phosphate_metabolism;Glycerophospholipid_metabolism;Nucleotide_interconversion;**Inositol_phosphate_metabolism*** | 27 |
| EMT_REGULATORS | 22 | Glycerophospholipid_metabolism;**Inositol_phosphate_metabolism***;Phosphatidylinositol_phosphate_metabolism;Selenoamino_acid_metabolism;Methionine_and_cysteine_metabolism | 36 |
| CYTOSKELETON_POLARITY | 5 | Glycerophospholipid_metabolism;**Inositol_phosphate_metabolism***;Phosphatidylinositol_phosphate_metabolism***** | 12 |
| E2F4_TARGETS | 11 | Starch_and_sucrose_metabolism;**Fructose_and_mannose_metabolism***;Folate_metabolism;Nucleotide_interconversion;Transport_extracellular | 12 |
| E2F1_TARGETS | 14 | **Fructose_and_mannose_metabolism***;Folate_metabolism;Eicosanoid_metabolism;Nucleotide_interconversion;Selenoamino_acid_metabolism;Methionine_and_cysteine_metabolism;**Inositol_phosphate_metabolism***;**Oxidative_phosphorylation***;Alkaloid_synthesis | 15 |
| AKT_MTOR | 3 | **Inositol_phosphate_metabolism***;Phosphatidylinositol_phosphate_metabolism;Glutathione_metabolism | 4 |
| PI3K_AKT_MTOR | 10 | Phosphatidylinositol_phosphate_metabolism;**Inositol_phosphate_metabolism***;Arginine_and_Proline_Metabolism | 13 |
| CASPASES | 3 | Glycerophospholipid_metabolism;Exchange/demand_reaction;Nucleotide_interconversion | 3 |
| ECM | 5 | **Inositol_phosphate_metabolism*** | 5 |
| TNF_RESPONSE | 13 | Vitamin_B2_metabolism;Glutamate_metabolism;Miscellaneous;Glutathione_metabolism;Arachidonic_acid_metabolism;Exchange/demand_reaction;Vitamin_C_metabolism | 15 |
| E2F3_TARGETS | 5 | Folate_metabolism;Nucleotide_interconversion | 5 |
| MAPK | 8 | Tetrahydrobiopterin_metabolism;**Inositol_phosphate_metabolism***;Phosphatidylinositol_phosphate_metabolism;Glycerophospholipid_metabolism;Exchange/demand_reaction | 11 |
| MOMP_REGULATION | 1 | **Fructose_and_mannose_metabolism***;Aminosugar_metabolism;**Glycolysis/gluconeogenesis*** | 3 |
| WNT_CANONICAL | 8 | Transport_lysosomal;Phosphatidylinositol_phosphate_metabolism;Glycerophospholipid_metabolism;Tetrahydrobiopterin_metabolism;**Inositol_phosphate_metabolism***;**Oxidative_phosphorylation*** | 14 |
| HEDGEHOG | 10 | Tetrahydrobiopterin_metabolism  **Inositol_phosphate_metabolism***;Phosphatidylinositol_phosphate_metabolism | 13 |
| E2F6_TARGETS | 5 | **Oxidative_phosphorylation***;Nucleotide_interconversion | 5 |
| RB | 1 | Lysine_metabolism | 1 |
| E2F6 | 2 | Lysine_metabolism | 2 |
| E2F2_TARGETS | 1 | Folate_metabolism | 1 |
| E2F4 | 1 | Lysine_metabolism | 1 |

**Additional file 6. Crosstalk between ACSN modules and ReconMap 2.0 metabolic pathways.** Using the composition of each module and subsystem (Additional files 2 and 3) and the list of the 252 shared proteins between both maps, modules and subsystems having common proteins were linked together. Thus, a crosstalk between both maps has been uncovered with the number of shared proteins showed here. *Modules and subsystems in bold are the result of an Enrichment Analysis of the 252 common proteins between ACSN and ReconMap 2.
