# Supplementary material for: Metabolic and signalling network maps integration: application to cross-talk studies and omics data analysis in cancer
Source: BMC Bioinformatics. 2019 Apr 18;20(Suppl 4):140. doi: 10.1186/s12859-019-2682-z (PMC6471697; doi:10.1186/s12859-019-2682-z)
Supplement: Supplementary file 6 — Enrichment analysis of common proteins between ACSN modules and ReconMap 2.0 metabolic pathways. Enrichment Analysis of the 252 common proteins between ACSN and ReconMap 2.0 was performed using the Survival Function Method on the union of proteins of both maps. The p-value is obtained through a standard Hypergeometric test on modules containing at least 10 proteins using a Bonferroni adjustment for the multiple testing correction. (DOCX 14 kb) [file 12859_2019_2682_MOESM6_ESM.docx]

| **Module/pathway name** | **Number of genes in module/pathway** | **Module/pathway total size** | **P-value** |
| --- | --- | --- | --- |
| **ACSN** | | | |
| MITOCH_METABOLISM | 202 | 382 | 1.48e-161 |
| APOPTOSIS_GENES | 46 | 190 | 5.01e-12 |
| **ReconMap 2** | | | |
| Oxidative_phosphorylation | 72 | 96 | 1.41e-62 |
| Citric_acid_cycle | 23 | 28 | 2.86e-20 |
| Glycolysis/gluconeogenesis | 35 | 81 | 1.59e-17 |
| Inositol_phosphate_metabolism | 25 | 65 | 8.30e-11 |
| Fructose_and_mannose_metabolism | 14 | 31 | 8.97e-07 |
| Pentose_phosphate_pathway | 13 | 22 | 4.10e-08 |

**Additional file 7. Enrichment analysis of common proteins between ACSN modules and ReconMap 2.0 metabolic pathways.** Enrichment Analysis of the 252 common proteins between ACSN and ReconMap 2.0 was performed using the Survival Function Method on the union of proteins of both maps. The p-value is obtained through a standard Hypergeometric test on modules containing at least 10 proteins using a Bonferroni adjustment for the multiple testing correction.
