# Supplementary material for: Metabolic and signalling network maps integration: application to cross-talk studies and omics data analysis in cancer
Source: BMC Bioinformatics. 2019 Apr 18;20(Suppl 4):140. doi: 10.1186/s12859-019-2682-z (PMC6471697; doi:10.1186/s12859-019-2682-z)
Supplement: Supplementary file 7 — Regulations of reactions in ReconMap 2.0 subsystems by proteins found in ACSN modules. The information encoded in the annotations of reactions and entities on the CellDesigner XML files was analysed and the correspondence between shared proteins in ACSN modules to regulated reactions in ReconMap 2.0 subsystems was retrieved and quantified. Number of ReconMap 2.0 reaction regulated by a subset of shared proteins is shown. (DOCX 17 kb) [file 12859_2019_2682_MOESM7_ESM.docx]

| **ACSN Modules** | **ReconMap 2 metabolic pathways** | **Number of catalyzed reactions in ReconMap 2 subsystems** |
| --- | --- | --- |
| TNF response | Vitamin B2 metabolism  Glycerophospholipid metabolism | 3 |
| Caspase | Nucleotide Interconversion | 4 |
| Hedgehog | Inositol phosphate metabolism | 6 |
| MapK | Inositol phosphate metabolism  Glycerophospholipid metabolism | 9 |
| PI3K AKT mTOR | Inositol phosphate metabolism  Arginine and Proline Metabolism | 13 |
| WNT non canonical | Inositol phosphate metabolism  Glycolysis/gluconeogenesis  Oxidative phosphorylation | 7 |
| WNT canonical | Inositol phosphate metabolism  Nucleotide Interconversion | 18 |
| EMT Motility | Inositol phosphate metabolism  Glycerophospholipid metabolism  Phosphatidylinositol phosphate metabolism  Methionine and cysteine metabolism  Selenoamino acid metabolism | 28 |
| Cellcycle | Transport extracellular mainly of Amino Acids  Nucleotide Interconversion  Folate metabolism  Amino Acids metabolism  Oxidative phosphorylation  Sugar metabolism | 69 |
| Apoptosis genes | Energy metabolism - sugar/amino/citric acid…  Extracellular transport of Amino Acids  Heme metabolism  Inositol phosphate metabolism | 102 |
| Mitochondrial metabolism | Energy metabolism - sugar/amino/citric acid…  Extracellular transport in Energy metabolism  Fatty acid synthesis  Nucleotide Interconversion  Amino Acids metabolism  Transport mitochondrial  Heme metabolism | 295 |

**Additional file 4. Regulations of reactions in ReconMap 2.0 subsystems by proteins found in ACSN modules.**

The information encoded in the annotations of reactions and entities on the CellDesigner XML files was analyzed and the correspondence between shared proteins in ACSN modules to regulated reactions in ReconMap 2.0 subsystems was retrieved and quantified. Number of ReconMap 2.0 reaction regulated by a subset of shared proteins is shown.
